# Supplementary material for: The Construction and Exploration of a Comprehensive MicroRNA Centered Regulatory Network in Foxtail Millet (Setaria italica L.)
Source: Front Plant Sci. 2022 May 6;13:848474. doi: 10.3389/fpls.2022.848474 (PMC9121102; doi:10.3389/fpls.2022.848474)
Supplement: Supplementary file 5 [file Data_Sheet_5.zip › Sit-miR393b_Seita.7G328000.1_195_TPlot.pdf]

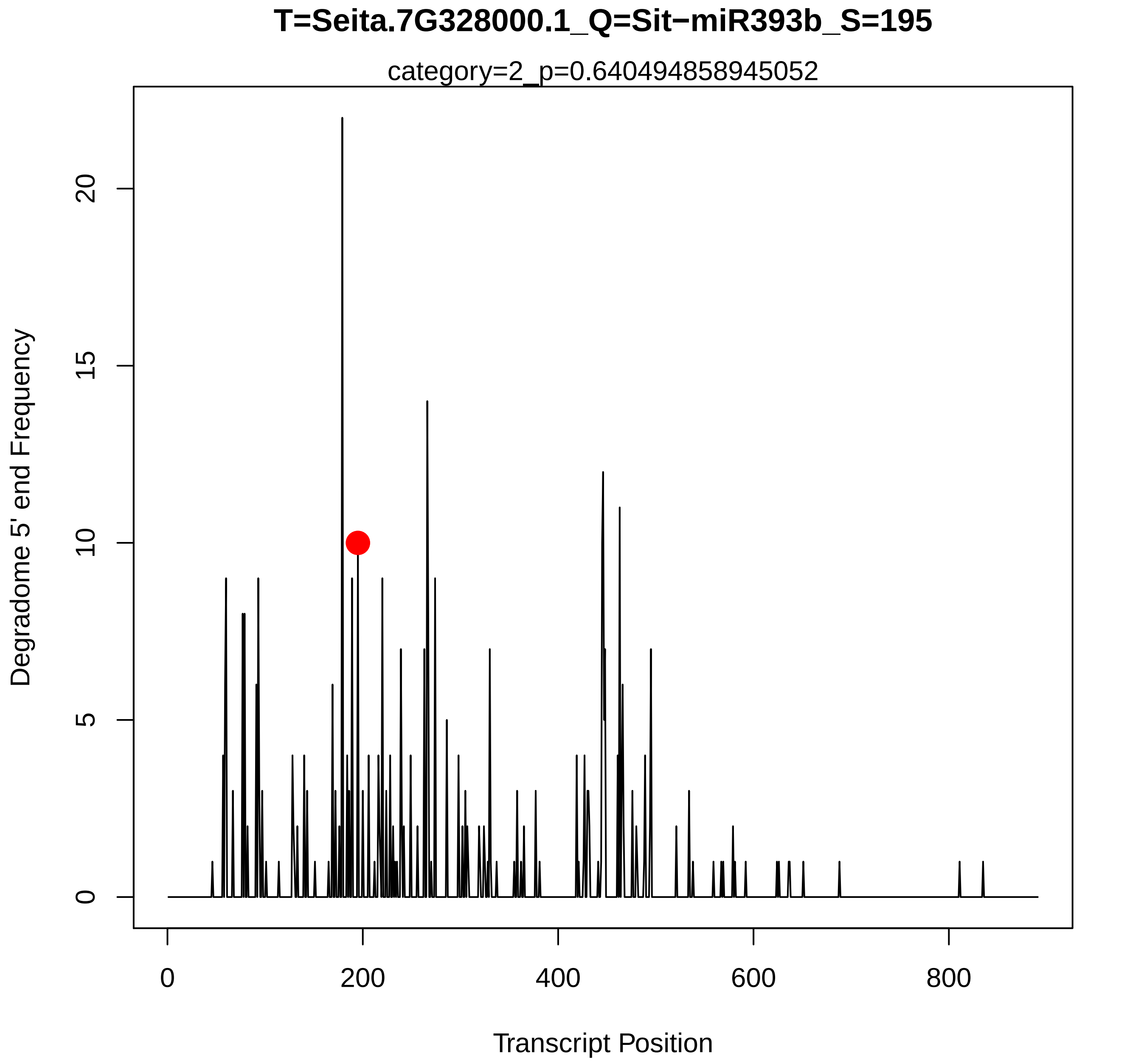
T=Seita.7G328000.1_Q=Sit−miR393b_S=195
category=2_p=0.640494858945052
Degradome 5' end Frequency
0
5
10
15
20
0
200
400
600
800
Transcript Position
